# Temporal and regional variation in the use of biologic and targeted synthetic DMARDs for rheumatoid arthritis: a nationwide cohort study

**DOI:** 10.1093/rheumatology/keae607

**Published:** 2024-11-01

**Authors:** Mark D Russell, Zijing Yang, Niamh Dooley, Mark Gibson, Benjamin Zuckerman, Maryam A Adas, Edward Alveyn, Samir Patel, Katie Bechman, Elizabeth Price, Sarah Gallagher, Callum Coalwood, Andrew P Cope, Sam Norton, James B Galloway

**Affiliations:** Centre for Rheumatic Diseases, King’s College London, London, UK; Centre for Rheumatic Diseases, King’s College London, London, UK; Centre for Rheumatic Diseases, King’s College London, London, UK; Centre for Rheumatic Diseases, King’s College London, London, UK; Centre for Rheumatic Diseases, King’s College London, London, UK; Centre for Rheumatic Diseases, King’s College London, London, UK; Centre for Rheumatic Diseases, King’s College London, London, UK; Centre for Rheumatic Diseases, King’s College London, London, UK; Centre for Rheumatic Diseases, King’s College London, London, UK; Department of Rheumatology, Great Western Hospital NHS Foundation Trust, Swindon, UK; National Early Inflammatory Arthritis Audit, British Society for Rheumatology, London, UK; National Early Inflammatory Arthritis Audit, British Society for Rheumatology, London, UK; Centre for Rheumatic Diseases, King’s College London, London, UK; Centre for Rheumatic Diseases, King’s College London, London, UK; Centre for Rheumatic Diseases, King’s College London, London, UK

**Keywords:** rheumatoid arthritis, biologic, JAK inhibitor, NEIAA, audit, variation, NICE, region

## Abstract

**Objective:**

The objective of this study was to evaluate temporal and regional variation in biologic and targeted synthetic DMARD (b/tsDMARD) initiation for RA in England and Wales.

**Methods:**

An observational cohort study was conducted for people with RA enrolled in the National Early Inflammatory Arthritis Audit (NEIAA) between May 2018 and April 2022 who had 12-month follow-up data available. Temporal trends in escalation to b/tsDMARDs within 12 months of initial rheumatology assessment were explored, including comparisons before and after publication (July 2021) of national guidelines that lowered the threshold for b/tsDMARD initiation to include moderate-severity RA. Case-mix–adjusted, mixed-effects regression was used to evaluate regional and hospital-level variation in b/tsDMARD initiation.

**Results:**

Of 6098 RA patients with available follow-up, 508 (8.3%) initiated b/tsDMARDs within 12 months of initial assessment. b/tsDMARD escalation increased marginally towards the end of the study period (9.2% in May 2021/22); however, no significant differences were evident after guidelines were published permitting b/tsDMARD use for moderate-severity RA. The proportion of individuals escalated to b/tsDMARDs varied considerably between regions, ranging from 5.1% in Wales to 10.7% in North-West England. Following case-mix adjustment, the intraclass correlation (ICC) for hospitals within regions was 0.17, compared with a between-region ICC of 0.0, suggesting that the observable regional variation reflected hospital-level differences rather than systematic differences between regions themselves.

**Conclusion:**

There is marked variation in escalation to b/tsDMARDs for people newly diagnosed with RA throughout England and Wales, despite a universal health-care system. These disparities must be addressed if we are to deliver equitable access to b/tsDMARDs, regardless of geography.

Rheumatology key messages

- There is substantial geographical variation in b/tsDMARD initiation for rheumatoid arthritis throughout England and Wales.
- This variation is driven primarily by differences between individual hospitals, rather than systematic differences between regions.
- b/tsDMARD initiation remained stable in the year after the publication of the NICE TA715 guideline for moderate-severity RA.

## Introduction

Biologic and targeted synthetic DMARDs (b/tsDMARDs) have transformed outcomes for people who have active RA despite treatment with conventional synthetic DMARDs (csDMARDs). Prompt escalation to b/tsDMARDs in these patients can improve disease control and prevent morbidity [1].

Until recently in England and Wales, access to b/tsDMARDs for RA via the publicly funded National Health Service (NHS) was restricted to people with severe disease, defined as a DAS at 28 joints (DAS28) of >5.1 despite intensive treatment with two or more csDMARDs [2]. The falling acquisition costs of b/tsDMARDs now means that it is cost-effective for a greater range of patients to benefit from treatment with b/tsDMARDs. In July 2021, the National Institute for Health and Care Excellence (NICE) lowered the threshold for escalation to b/tsDMARDs to include people with RA who have moderate disease activity (DAS28 3.2–5.1) despite csDMARD therapy [3]. It remains unclear to what extent this guidance has impacted on the proportion of individuals with RA who are escalated to b/tsDMARDs early in the course of their disease.

The Healthcare Quality Improvement Partnership (HQIP) in England and Wales commissioned the National Early Inflammatory Arthritis Audit (NEIAA) in 2018, with the objective of improving the quality of care provided to people with RA and other inflammatory rheumatic diseases. NEIAA is a mandatory national audit programme, through which data are collected prospectively on people with new inflammatory arthritis diagnoses referred to rheumatology services in England and Wales [4]. These data have been used to highlight unwarranted variation in inflammatory arthritis care between regions and hospitals in England and Wales, including delays in diagnosis and the initiation of csDMARDs [5, 6].

Our objective was to use NEIAA data to evaluate what proportion of individuals with RA are escalated to b/tsDMARDs within 12 months of diagnosis, and to describe how this has varied over time and by region.

## Methods

### Data source and study sample

We conducted an observational cohort study using data from NEIAA, a national clinical audit programme that facilitates benchmarking of care quality for people with early inflammatory arthritis (EIA) diagnoses in England and Wales [4]. Since 8 May 2018, NHS providers of rheumatology services in England and Wales have been mandated to submit data to NEIAA on all patients aged 16 years or over who are newly referred with suspected EIA diagnoses. In this study, we included all people with diagnoses of RA (defined by the treating clinician on the basis of clinical judgement) who were enrolled in NEIAA between 8 May 2018, and 30 April 2022, who were eligible for follow-up, and who had 12-month follow-up data available.

### Outcome measures

The outcome measure of interest was escalation to a b/tsDMARD (TNF inhibitor, rituximab, abatacept, IL-6 inhibitor or JAK inhibitor) for the treatment of RA within 12 months of a patient’s first rheumatology appointment. This information was recorded as a binary outcome by the treating clinician at 12 months (±2 months) following the initial rheumatology visit. No additional information is collected on whether this represents the first or subsequent b/tsDMARD for an individual patient.

### Baseline characteristics

Baseline characteristics were tabulated for individuals who initiated b/tsDMARDs by 12 months, and were presented without inferential statistics for the overall cohort and separated by region. To facilitate comparisons with the study cohort, baseline characteristics were also presented for RA patients enrolled in NEIAA who did *vs* did not have 12-month follow-up data available, and for RA patients enrolled in NEIAA who initiated *vs* did not initiate b/tsDMARDs.

### Temporal analyses

Two-way plots were used to display temporal changes in the proportion of individuals who were escalated to b/tsDMARDs over the study period. Separate plots were used to show the proportion of individuals commenced on TNF inhibitors *vs* other mode-of-action b/tsDMARDs at 12 months. Interrupted time-series analyses (ITSA) were used to estimate the impact of the NICE TA715 guideline (‘Adalimumab, etanercept, infliximab and abatacept for treating moderate rheumatoid arthritis after conventional DMARDs have failed’ [3]) on the proportion of patients escalated to b/tsDMARDs. Trends were compared before and after the publication of this guideline (14 July 2021) using single-group ITSA [7]. In these models, single time-points represented the mean proportion of individuals escalated to b/tsDMARDs within 12 months of initial assessment, averaged over consecutive 3-month periods. Autocorrelation between observation periods was accounted for using Newey-West standard errors with three lags [7].

### Regional analyses

Logistic mixed-effects regression models were used to explore the effect of region and hospital on the probability of individuals being escalated to b/tsDMARDs within 12 months of assessment. The treating Hospital Trust is an organizational unit of one or more hospitals serving a local population in England. In Wales, these organizational units are represented as local health boards in NEIAA. Hospital Trusts/local health boards and the wider region (defined in NEIAA as the seven NHS England commissioning regions and Wales [8]) were included as random intercepts in three-level models. The observed probability of initiating b/tsDMARDs was presented by region, alongside case-mix–adjusted empirical Bayes probability estimates from the mixed-effects models, whereby patient-level factors were held constant across regions. Empirical Bayes estimates from logistic mixed-effects models incorporate information from higher levels (e.g. region) to inform estimates at lower levels (e.g. hospital), effectively ‘shrinking’ estimates towards the higher-level (e.g. regional) means. This leads to more stable and less variable estimates, particularly when individual-level data are sparse or noisy (e.g. where relatively few people are enrolled from some hospitals). Patient-level factors used for case-mix adjustment were selected a priori from the variables collected in NEIAA on the basis of whether they were felt to be potentially important predictors of b/tsDMARD initiation: age; sex (recorded by clinicians from patients’ medical records); index of multiple deprivation (IMD; an area-level, composite score of socioeconomic position); rheumatic disease comorbidity index (RDCI; a scoring system designed to quantify the total burden of comorbidity in people with RA [9]); and baseline disease severity (DAS28). Regional variation was depicted graphically using geographical heat maps, shown before and after case-mix adjustment. This was presented alongside the probability of b/tsDMARD initiation within individual Hospital Trusts in each region, estimated using empirical Bayes means. The intraclass correlations (ICCs), representing the proportion of the total variance attributable to differences between regions and differences between Hospital Trusts, were presented from unadjusted and case-mix–adjusted models. ICC estimates from case-mix–adjusted models indicate the proportion of the residual variance (i.e. not explained by case-mix) that is attributable to the level of clustering.

Exploratory analyses were performed to investigate hospital-level factors that might have influenced the initiation of b/tsDMARDs, as follows: the number of annual rheumatology appointments in each Hospital Trust (obtained from publicly available, aggregate-level, hospital outpatient activity data from NHS England [10] and StatsWales [11]; the number of whole-time equivalent (WTE) rheumatology consultants and WTE rheumatology nurses in each Hospital Trust (collected via NEIAA); the ratio of rheumatology consultants and nurses to the number of annual appointments (calculated using the above data sources); and the presence or absence of dedicated EIA clinics within each rheumatology service (collected via NEIAA). Associations between these factors and the proportion of individuals escalated to b/tsDMARDs within each Hospital Trust were estimated using linear regression. Additional analyses were performed to explore regional variation in the choice of b/tsDMARD at 12 months (categorized into TNF inhibitors *vs* other mode-of-action b/tsDMARDs), using logistic mixed-effects regression models with and without case-mix-adjustment, as described above.

All statistical analyses were conducted in Stata version 18. No correction was made for multiple hypothesis testing.

### Ethical approval

Approval to conduct this research using NEIAA was obtained from HQIP. No further ethical approval was required.

## Results

Of 14 233 people with new RA diagnoses enrolled in NEIAA between May 2018 and April 2022, 6098 (42.8%) had 12-month follow-up data available on whether b/tsDMARDs were initiated (Supplementary Fig. S1, available at *Rheumatology* online). The baseline characteristics of people with RA who had available follow-up data were comparable with those without available follow-up data (Supplementary Table S1, available at *Rheumatology* online).

Of 6098 individuals with available follow-up data, 508 (8.3%) were escalated to b/tsDMARDs within 12 months of initial rheumatology assessment (Supplementary Table S2, available at *Rheumatology* online). At 12 months, TNF inhibitors were the most frequently recorded b/tsDMARD [403/508 (79.3%)], followed by JAK inhibitors [48/508 (9.5%)], rituximab [32/508 (6.3%])], IL-6 inhibitors [16/508 (3.2%)] and abatacept [9/508 (1.8%)].

**Figure 1.**
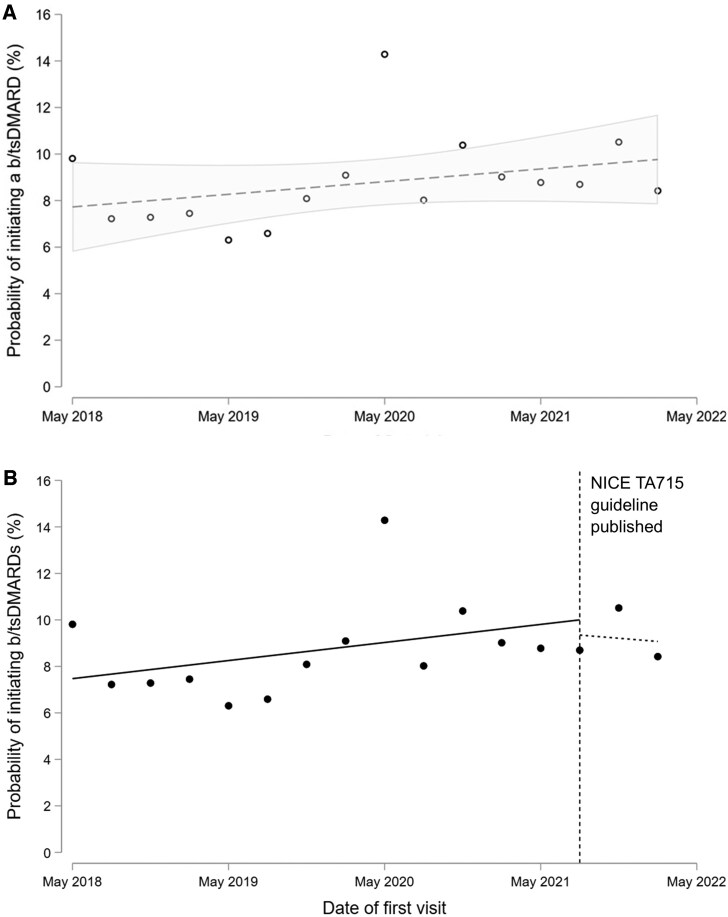
Temporal trends in escalation to b/tsDMARDs for individuals with new RA diagnoses enrolled in NEIAA. Single time-points represent mean proportions of individuals escalated to b/tsDMARDs within 12 months of initial rheumatology assessment, averaged over consecutive 3-month time-periods. In Panel A (top), a linear trend-line is shown with 95% CIs (shaded area). In Panel B (bottom), an interrupted time-series analysis compares trends in the periods before *vs* after the publication of the NICE TA715 guideline (July 2021; dashed vertical line), which lowered the threshold for b/tsDMARD initiation to include people with DAS28 scores between 3.2 and 5.1. Trends before the publication of NICE guidance: 0.78% increase per year; trend after the publication of NICE guidance: 0.55% decrease per year; relative change: −1.32% per year (*P* = 0.43). b/tsDMARD: biologic/targeted synthetic DMARD; DAS28: DAS at 28 joints; NICE: National Institute for Health and Care Excellence

The proportion of individuals escalated to b/tsDMARDs increased modestly towards the end of the study period (Fig. 1A): 8.1% of individuals initially assessed between May 2018 and April 2019 were escalated to b/tsDMARDs within 12 months; 7.2% of individuals initially assessed between May 2019 and May 2020; 9.5% between May 2020 and May 2021; and 9.2% between May 2021 and May 2022. Comparable temporal trends were observed for TNF inhibitors and other mode-of-action b/tsDMARDs (Supplementary Fig. S2, available at *Rheumatology* online).

There was a temporary spike in b/tsDMARD initiation for individuals assessed between May 2020 and July 2020, with 14.3% of patients escalated to b/tsDMARDs within 12 months of this period (Fig. 1A). However, this must be interpreted in the context of fewer individuals being enrolled in NEIAA during this time period due to the temporary pause in mandatory data collection during the early COVID-19 pandemic (Supplementary Table S3, available at *Rheumatology* online) [12]. In interrupted time-series analyses, there were no significant differences in trends in the initiation of b/tsDMARDs comparing the periods before and after the publication of the NICE TA715 guideline in July 2021, which lowered the threshold for b/tsDMARD initiation to include treatment of moderate-severity RA (Fig. 1B).

Of 5590 individuals not escalated to b/tsDMARDs within 12 months, 4975 (89.0%) had data available on their DAS28-ESR or DAS28-CRP at 12 months following initial assessment. Of these individuals, 429 (8.6%) had a DAS28-ESR or DAS28-CRP of >5.1; 1391 (28.0%) had a DAS28-ESR or DAS28-CRP between 3.2 and 5.1; and 3155 (63.4%) had a DAS28-ESR and/or DAS28-CRP of <3.2. These data were comparable when broken down by year of initial assessment (Supplementary Table S4, available at *Rheumatology* online), including after the introduction of the NICE TA715 guideline.

**Figure 2.**
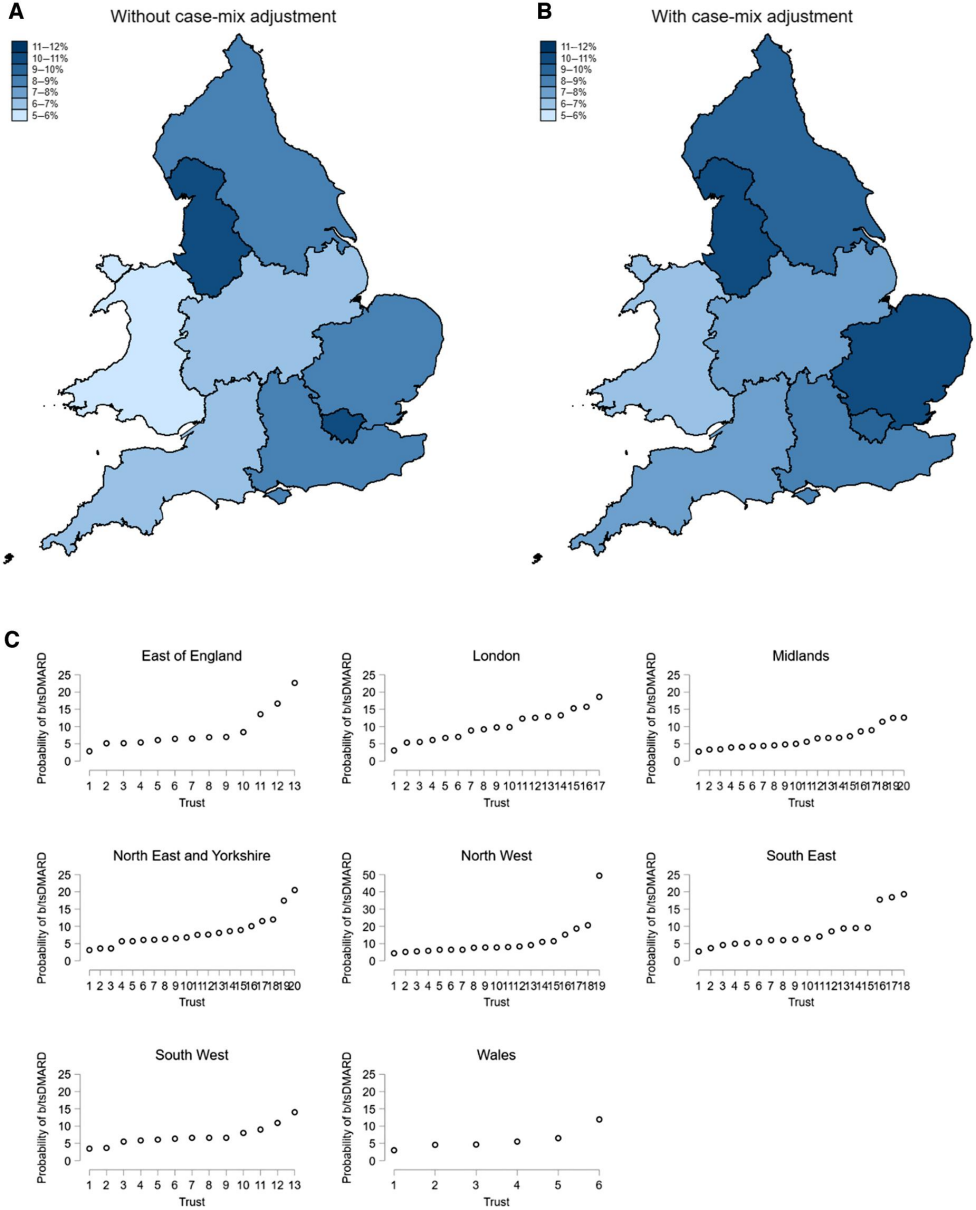
Variation by region (Panels A and B) and Hospital Trust (Panel C) in the proportion of individuals with RA who were escalated to b/tsDMARDs within 12 months of initial rheumatology assessment. Regional heat maps are shown before (Panel A) and after case-mix adjustment (Panel B). Case-mix adjustment was performed by holding the following variables at constant levels (specified in brackets) across regions: age (50 years); sex (female); index of multiple deprivation (middle tertile); rheumatic disease comorbidity index (one comorbidity); DAS28 at baseline (5.1). Variation in b/tsDMARD initiation between individual Hospital Trusts within each region is shown in Panel C (in Wales, these organizational units are represented as local health boards in NEIAA). b/tsDMARD: biologic/targeted synthetic DMARD; DAS28: DAS at 28 joints; NEIAA: National Early Inflammatory Arthritis Audit

There was marked regional variation in the observed proportion of individuals who were escalated to b/tsDMARDs (Fig. 2A; Supplementary Fig. S3A, available at *Rheumatology* online). This varied from 5.1% of individuals assessed in Wales (95% CI 2.9–8.2%), to 10.1% of individuals assessed in London (95% CI 7.9–12.6%) and 10.7% in North-West England (95% CI 8.7–13.0%). There were notable differences in case-mix between these regions (Table 1), with individuals assessed in Wales having a greater comorbidity burden at presentation, particularly chronic lung disease and cancer, relative to individuals assessed in regions of England. Disease severity at baseline was comparable between regions, as was the time to initial diagnosis, and the proportion of individuals who initiated csDMARDs within 3 months of initial assessment. Following adjustment for case-mix, including age, sex, socioeconomic status, comorbidity burden, and disease severity at baseline, there was a reduction in the magnitude of difference in escalation to b/tsDMARDs between regions (Fig. 2B; Supplementary Fig. S3B, available at *Rheumatology* online); however, the underlying patterns remained similar, with individuals in Wales least likely to be initiated on b/tsDMARDs (case-mix–adjusted probability: 6.7%; 95% CI 4.2–10.2%), while individuals in North-West England remained most likely to be initiated on b/tsDMARDs (adjusted probability: 10.5%; 95% CI 8.5–12.8%). The regional variation in b/tsDMARD escalation during each year of the study period is shown in Supplementary Fig. S4, available at *Rheumatology* online.

**Table 1.** Escalation to b/tsDMARDs and baseline characteristics of individuals with RA diagnoses enrolled in NEIAA, separated by region

	Total	East of England	London	Midlands	North East and Yorkshire	North West England	South East England	South West England	Wales
	*N* = 6098	*N* = 796	*N* = 685	*N* = 1114	*N* = 855	*N* = 831	*N* = 882	*N* = 639	*N* = 296
Outcome									
b/tsDMARD initiated	508 (8.3%)	69 (8.7%)	69 (10.1%)	71 (6.4%)	76 (8.9%)	89 (10.7%)	77 (8.7%)	42 (6.6%)	15 (5.1%)
No b/tsDMARD initiated	5590 (91.7%)	727 (91.3%)	616 (89.9%)	1043 (93.6%)	779 (91.1%)	742 (89.3%)	805 (91.3%)	597 (93.4%)	281 (94.9%)
b/tsDMARD at 12 months									
TNF inhibitor	403 (79.3%)	60 (87.0%)	60 (87.0%)	57 (80.3%)	54 (71.1%)	75 (84.3%)	54 (70.1%)	34 (81.0%)	9 (60.0%)
Other mode-of-action b/tsDMARD	105 (20.7%)	9 (13.0%)	9 (13.0%)	14 (19.7%)	22 (28.9%)	14 (15.7%)	23 (29.9%)	8 (19.0%)	6 (40.0%)
Age, years [mean (s.d.)]	59.2 (14.9)	60.6 (14.5)	55.4 (15.5)	58.9 (14.6)	58.8 (14.4)	59.5 (14.4)	59.0 (15.6)	61.4 (14.8)	60.7 (14.3)
Sex									
Male	2186 (35.8%)	287 (36.1%)	218 (31.8%)	436 (39.1%)	306 (35.8%)	284 (34.2%)	327 (37.1%)	229 (35.8%)	99 (33.4%)
Female	3912 (64.2%)	509 (63.9%)	467 (68.2%)	678 (60.9%)	549 (64.2%)	547 (65.8%)	555 (62.9%)	410 (64.2%)	197 (66.6%)
Ethnicity									
White	5215 (86.2%)	733 (92.1%)	350 (51.1%)	954 (85.6%)	799 (93.5%)	779 (94.0%)	694 (83.3%)	617 (96.6%)	289 (97.6%)
Black, Asian, Mixed or Other	832 (13.8%)	63 (7.9%)	335 (48.9%)	160 (14.4%)	56 (6.5%)	50 (6.0%)	139 (16.7%)	22 (3.4%)	7 (2.4%)
Not known	51	0	0	0	0	2	49	0	0
IMD decile									
1–3 (least deprived)	1563 (26.6%)	306 (39.6%)	116 (17.2%)	240 (23.9%)	141 (16.7%)	92 (11.2%)	440 (53.1%)	169 (26.8%)	59 (19.9%)
4–7	2457 (41.8%)	288 (37.3%)	346 (51.4%)	416 (41.4%)	224 (26.5%)	309 (37.5%)	268 (32.3%)	438 (69.4%)	168 (56.8%)
8–10 (most deprived)	1855 (31.6%)	178 (23.1%)	211 (31.4%)	348 (34.7%)	480 (56.8%)	424 (51.4%)	121 (14.6%)	24 (3.8%)	69 (23.3%)
Not known	223	24	12	110	10	6	53	8	0
Comorbidity burden (RDCI)									
None	3377 (55.5%)	422 (53.0%)	369 (53.9%)	617 (55.5%)	466 (54.8%)	439 (53.0%)	553 (62.7%)	381 (59.8%)	130 (43.9%)
One	1331 (21.9%)	176 (22.1%)	142 (20.7%)	265 (23.8%)	185 (21.7%)	192 (23.2%)	182 (20.6%)	129 (20.3%)	60 (20.3%)
Two or more	1380 (22.7%)	198 (24.9%)	174 (25.4%)	230 (20.7%)	200 (23.5%)	198 (23.9%)	147 (16.7%)	127 (19.9%)	106 (35.8%)
Not known	10	0	0	2	4	2	0	2	0
Lung disease									
No	5387 (88.5%)	696 (87.4%)	609 (88.9%)	997 (89.7%)	758 (89.1%)	724 (87.3%)	805 (91.3%)	565 (88.7%)	233 (78.7%)
Yes	701 (11.5%)	100 (12.6%)	76 (11.1%)	115 (10.3%)	93 (10.9%)	105 (12.7%)	77 (8.7%)	72 (11.3%)	63 (21.3%)
Not known	10	0	0	2	4	2	0	2	0
Cardiovascular disease									
No	5754 (94.5%)	752 (94.5%)	650 (94.9%)	1057 (95.1%)	792 (93.1%)	774 (93.4%)	858 (97.3%)	594 (93.2%)	277 (93.6%)
Yes	334 (5.5%)	44 (5.5%)	35 (5.1%)	55 (4.9%)	59 (6.9%)	55 (6.6%)	24 (2.7%)	43 (6.8%)	19 (6.4%)
Not known	10	0	0	2	4	2	0	2	0
Cancer									
No	5846 (96.0%)	759 (95.4%)	662 (96.6%)	1070 (96.2%)	820 (96.4%)	797 (96.1%)	848 (96.1%)	616 (96.7%)	274 (92.6%)
Yes	242 (4.0%)	37 (4.6%)	23 (3.4%)	42 (3.8%)	31 (3.6%)	32 (3.9%)	34 (3.9%)	21 (3.3%)	22 (7.4%)
Not known	10	0	0	2	4	2	0	2	0
Depression									
No	5656 (92.9%)	735 (92.3%)	649 (94.7%)	1028 (92.4%)	776 (91.2%)	754 (91.0%)	842 (95.5%)	607 (95.3%)	265 (89.5%)
Yes	432 (7.1%)	61 (7.7%)	36 (5.3%)	84 (7.6%)	75 (8.8%)	75 (9.0%)	40 (4.5%)	30 (4.7%)	31 (10.5%)
Not known	10	0	0	2	4	2	0	2	0
Smoking status									
Current smoker	1189 (21.0%)	172 (23.1%)	124 (20.1%)	229 (22.7%)	186 (22.4%)	161 (20.4%)	155 (20.2%)	103 (16.7%)	59 (20.3%)
Ex-smoker	1820 (32.1%)	261 (35.0%)	128 (20.8%)	308 (30.6%)	274 (33.1%)	283 (35.9%)	223 (29.0%)	219 (35.6%)	124 (42.8%)
Never smoked	2652 (46.8%)	313 (42.0%)	364 (59.1%)	470 (46.7%)	369 (44.5%)	344 (43.7%)	391 (50.8%)	294 (47.7%)	107 (36.9%)
Not known	437	50	69	107	26	43	113	23	6
RF or CCP positive	4308 (73.6%)	554 (72.2%)	521 (77.5%)	843 (78.2%)	612 (73.0%)	589 (73.7%)	492 (63.3%)	461 (73.4%)	236 (80.0%)
Not known	244	29	13	36	17	32	105	11	1
DAS28 at baseline, median [IQR]	5.1 (4.1,6.0)	4.9 (3.9,5.9)	5.1 (4.1,6.0)	5.1 (4.1,6.0)	5.2 (4.2,6.0)	5.2 (4.2,6.1)	5.1 (3.9,5.9)	5.0 (4.1,5.9)	5.0 (4.0,5.9)
Not known	265	22	15	65	15	72	55	19	2
Median time from referral to diagnosis, days (median [IQR])	17 (10,32)	18 (10,30)	16 (11,33)	19 (11,34)	16 (9,30)	20 (10,39)	16 (9,32)	17 (10,26)	18 (12,34)
Not known	29	0	0	6	10	5	6	1	1
csDMARD initiated by 3 months	5846 (95.9%)	769 (96.6%)	669 (97.7%)	1046 (93.9%)	816 (95.4%)	795 (95.7%)	843 (95.6%)	616 (96.4%)	292 (98.6%)
MTX initiated by 3 months	4296 (70.4%)	533 (67.0%)	422 (61.6%)	782 (70.2%)	604 (70.6%)	575 (69.2%)	632 (71.7%)	517 (80.9%)	231 (78.0%)
EULAR response at 3 months									
No response	1614 (30.4%)	204 (28.8%)	224 (34.7%)	311 (33.3%)	225 (28.8%)	171 (27.1%)	226 (30.6%)	173 (29.8%)	80 (28.4%)
Moderate response	1673 (31.6%)	203 (28.7%)	211 (32.7%)	300 (32.1%)	276 (35.4%)	226 (35.8%)	212 (28.7%)	168 (28.9%)	77 (27.3%)
Good response	2015 (38.0%)	301 (42.5%)	210 (32.6%)	324 (34.7%)	279 (35.8%)	235 (37.2%)	301 (40.7%)	240 (41.3%)	125 (44.3%)
Not known	796	88	40	179	75	199	143	58	14

Ethnicity has been grouped into two categories due to the small numbers of individuals from minority ethnicity groups within some regions and the potential for disclosure. EULAR response represents the response to treatment (e.g. csDMARDs) at 3 months following initial assessment by a rheumatologist. csDMARD: conventional synthetic DMARD; b/tsDMARD: biologic/targeted synthetic DMARD; IMD: index of multiple deprivation; IQR: interquartile range; RDCI: rheumatic disease comorbidity index; DAS28: DAS at 28 joints.

More variation in b/tsDMARD escalation was evident between individual Hospital Trusts within each region than between the regions themselves (Fig. 2C). Prior to case-mix adjustment, the ICC for Hospital Trusts within each region was 0.15 (95% CI 0.10–0.22), compared with a between-region ICC of 0.0048 (95% CI 0.00–0.27). This indicates that ∼15% of the variability in b/tsDMARD escalation is due to differences between hospitals, and that any observable regional variation reflects hospital-level differences rather than systematic differences between regions. Following case-mix adjustment, the ICC for Hospital Trusts within regions was 0.17 (95% 0.12–0.25), compared with a between-region ICC of 0.0. The probability of individual Hospital Trusts escalating individuals to b/tsDMARDs was not significantly associated with how many rheumatology appointments there were in each hospital on an annual basis; how well staffed the hospitals were in terms of rheumatology consultants and/or rheumatology nurse specialists; or whether hospitals had dedicated EIA clinics (Supplementary Table S5, available at *Rheumatology* online).

For people who were commenced on b/tsDMARDs for RA, Wales had the highest proportion of individuals prescribed b/tsDMARDs other than TNF inhibitors at 12 months (40.0%, compared with 13.0% to 29.9% throughout regions of England; Table 1). Following adjustment for case-mix differences between regions, including comorbidity burden, the variation in TNF inhibitor *vs* non-TNF inhibitor use between regions became less marked (Supplementary Fig. S5, available at *Rheumatology* online).

## Discussion

In this nationwide study, we demonstrated that 1 in 12 individuals with new diagnoses of RA were escalated to b/tsDMARDs within 12 months of initial rheumatology assessment. At a national level, this remained relatively stable between May 2018 and April 2022, with no observed detrimental impact of the COVID-19 pandemic, and no significant increase following the publication of guidelines that lowered the threshold for b/tsDMARD initiation to include people with moderate-severity RA. Underlying these national trends was marked geographic variation in the initiation of b/tsDMARDs. Individuals with RA assessed in Wales were 50% less likely to be initiated on b/tsDMARDs than individuals assessed in some regions of England. While regional variation persisted following case-mix adjustment, this was almost entirely due to variation between individual hospitals within each region, suggesting it is likely to reflect differences in prescribing practices between hospitals.

Targeted therapies have transformed outcomes for people with RA over the last two decades, but surprisingly few studies have reported the proportion of treatment-naïve RA patients who are escalated to b/tsDMARDs. Much of the existing literature stems from before the introduction of lower-cost biosimilars. In a US-based cohort study with data to 2013, 4.9% of patients with early untreated RA were initiated on bDMARDs during 12 months of follow-up [13]. In a US Veteran Affairs study with data to 2016, 12.6% of patients were initiated on bDMARDs for RA within 12 months of receiving their first MTX prescription [14]. In a Norwegian registry study with data to 2012, 19% of patients who had commenced their first DMARD for RA were initiated on bDMARDs during a mean follow-up of 2.6 years [15].

Our study benefitted from contemporaneous data on newly referred patients during a period when biosimilars are widely used. Using population-level data from nearly all hospitals in England and Wales, we showed that 8% of people newly referred with RA were escalated to b/tsDMARDs within 12 months of initial assessment. In the NHS of England and Wales, the cost of b/tsDMARDs is met by the health service if eligibility criteria (defined by NICE) are met. Until July 2021, NICE required a DAS28 of >5.1 despite treatment with two or more csDMARDs before funded access to b/tsDMARDs was provided [2]. In July 2021, NICE updated their recommendations to permit escalation to b/tsDMARDs to include people with moderate disease activity after failure of two or more csDMARDs [3]. In interrupted time-series analyses, with data up to 30 April 2022, we did not observe any meaningful impact of this guidance on escalation to b/tsDMARDs. One potential explanation for this finding is that insufficient time had elapsed by the end of our study for the updated guidance to have influenced prescribing. Another explanation could be that the number of people with newly diagnosed RA who meet the moderate RA eligibility criteria for b/tsDMARDs, but who would not otherwise have met the severe RA criteria, is relatively small (e.g. those with oligoarticular disease). Alternatively, it may be that clinicians are reserving b/tsDMARDs for people with severe disease activity, despite guidelines recommending a treat-to-target strategy with sustained remission or low disease activity as the target [16]. Of note, we showed that for individuals who were not escalated to b/tsDMARDs by 12 months, 37% had a DAS28 at 12 months that met the eligibility criteria for escalation to b/tsDMARDs. The majority of these patients had moderate-severity RA, which suggests that many more patients could be escalated to b/tsDMARDs if guidelines were followed.

One of the most striking findings was the degree to which b/tsDMARD initiation varied between individual regions and hospitals, despite England and Wales having a universal health-care system with clearly defined criteria for escalation to b/tsDMARDs. Individuals in Wales were 50% less likely to be escalated to b/tsDMARDs than those assessed in London or North-West England. This variation was not limited to Wales: individuals in the Midlands or South-West England were 40% less likely to be escalated to b/tsDMARDs than those in North-West England. After adjusting for case-mix differences, including comorbidity burden, the magnitude of variation between these regions reduced in size but did not disappear, suggesting that additional factors contribute to the inequity in b/tsDMARD utilization.

We observed marked variation in b/tsDMARD utilization between individual Hospital Trusts within each region, with some Hospital Trusts being several times more likely than others to escalate to b/tsDMARDs. We explored structural factors that might have influenced this hospital-level variation, including staffing levels, department size, and the presence of dedicated EIA services; none of which meaningfully impacted on rates of b/tsDMARD escalation. There are likely to be numerous prescriber-level factors that influence the decision to escalate to b/tsDMARDs, which we were unable to evaluate in our study due to limited data on prescribers and their characteristics. Data from countries including Canada, Sweden and the USA demonstrate the importance of physician preference on the decision to escalate to b/tsDMARDs, independent of patient-level factors [17–19]. In a study from Ontario, Canada, utilizing administrative data from 2002 to 2015 on adults with RA aged 67 years or older, prescriber-level factors explained 65% of the total variation in the time to initiation of b/tsDMARDs [19]. Physician-level factors that were associated with increased prescribing of b/tsDMARDs included more recent graduation from medical school and greater regional availability of rheumatologists, whereas prescribers in more rural locations were less likely to prescribe b/tsDMARDs [19].

In addition to geographic variation in the decision to escalate to b/tsDMARDs, we also observed regional variation in the choice of targeted therapy. In Wales, 40% of individuals who were escalated to b/tsDMARDs were prescribed b/tsDMARDs other than TNF inhibitors at 12 months, substantially more than any region of England. Numerous factors can influence the choice of targeted therapy, including physician preference, local prescribing pathways, and patient-level factors. In our study, comorbid lung disease and cancer were more prevalent at baseline in individuals assessed in Wales than in individuals assessed in other regions. Following case-mix adjustment, regional variation in the choice of targeted therapy became less marked, indicating the continued influence of these factors on b/tsDMARD choice, despite accumulating evidence supporting the safety of TNF inhibitors in people with cancer or interstitial lung disease [20–23].

Our study had a number of strengths, which include a nationwide, representative data source with mandatory participation from all rheumatology departments in England and Wales. NEIAA is one of the largest inception cohorts of EIA patients worldwide, enabling us to investigate predictors of b/tsDMARD use in a treatment-naïve cohort of RA patients. We used multi-level models with case-mix adjustment to explore factors associated with b/tsDMARD use. We were able to account for numerous potential confounders, including baseline disease severity, which are lacking from many of the administrative datasets that have been used to report b/tsDMARD utilization previously.

There were also limitations with our study. The relatively small number of people escalated to b/tsDMARDs precluded more granular analyses of b/tsDMARD choice. Limited data meant that it was not possible to quantify the proportion of variability due to differences between prescribers, which is likely to explain much of the variation between hospitals. We were also unable to explore the influence of patient choice (e.g. patients being offered b/tsDMARDs but declining to commence them). While it is mandatory for hospitals in England and Wales to participate in NEIAA, the audit still relies upon clinician engagement and resource availability, which potentially introduces sampling bias. The number of individuals in Wales who were enrolled in NEIAA was lower than in regions of England, which needs to be considered when interpreting the findings from our study. While all patients in this cohort were eligible for follow-up, only 43% had 12-month follow-up data available. Although baseline characteristics were comparable between individuals with and without follow-up data, it remains possible that there were systematic differences between those followed-up *vs* not followed-up (e.g. in the likelihood of escalation to b/tsDMARDs). Data on patients cared for outside of the NHS (e.g. the private sector) are not routinely captured in NEIAA, which represents a small minority of health-care delivery in the UK. b/tsDMARD initiation in NEIAA is captured as a binary outcome at 12 months after initial rheumatology assessment; however, no data were available on the time taken to initiate a b/tsDMARD, which precluded time-to-event analyses, or whether b/tsDMARDs were initiated beyond this point. One must also consider the potential for unmeasured confounding, residual confounding due to missing data, and measurement error in variables such as smoking status.

In conclusion, we have demonstrated marked regional disparity in the escalation to b/tsDMARDs for people with RA in England and Wales, despite the presence of a universal health-care system in these countries. Underlying this regional disparity in b/tsDMARD use was substantial variation between individual hospitals within regions. Of individuals not commenced on b/tsDMARDs, 37% would have been eligible for escalation to b/tsDMARDs based upon their disease severity scores at 12 months, suggesting many more patients could benefit from these treatments. We need a better understanding of the drivers behind these inequities in care, including the influence of prescriber-level factors in the decision to escalate to b/tsDMARDs. Addressing these factors will enable us to improve access to b/tsDMARDs for people with RA, regardless of geography.

## Supplementary Material

keae607_Supplementary_Data

## Data Availability

Data access requests can be made through the Healthcare Quality Improvement Partnership.
