# Supplementary material for: Temporal and regional variation in the use of biologic and targeted synthetic DMARDs for rheumatoid arthritis: a nationwide cohort study
Source: Rheumatology (Oxford). 2024 Nov 1;64(5):2432–41. doi: 10.1093/rheumatology/keae607 (PMC12048046; doi:10.1093/rheumatology/keae607)
Supplement: keae607_Supplementary_Data [file keae607_supplementary_data.docx]

**Supplementary Appendix Contents**

[**Supplementary Figure S1.** Flowchart of study cohorts. 2](#_Toc179625986)

[**Supplementary Figure S2.** Temporal trends in the proportion of individuals with RA who were commenced on TNF inhibitors vs. other mode-of-action b/tsDMARDs at 12 months after the initial rheumatology assessment. 3](#_Toc179625987)

[**Supplementary Figure S3.** Regional variation in the proportion of individuals in NEIAA with new RA diagnoses who were escalated to b/tsDMARDs within 12 months of initial rheumatology assessment. 4](#_Toc179625988)

[**Supplementary Figure S4.** Temporal and regional variation in the proportion of individuals with RA who were escalated to b/tsDMARDs within 12 months. 5](#_Toc179625989)

[**Supplementary Figure S5.** Regional variation in the proportion of individuals initiating b/tsDMARDs within 12 months of initial rheumatology assessment who were prescribed TNF inhibitors. 6](#_Toc179625990)

[**Supplementary Table S1**. Baseline characteristics of individuals with RA enrolled in NEIAA who had available 12-month follow-up data, compared with individuals with RA without available follow-up data. 7](#_Toc179625991)

[**Supplementary Table S2**. Baseline characteristics of individuals with RA who initiated b/tsDMARDs within 12 months of initial rheumatology assessment, compared with individuals who did not initiate b/tsDMARDs. 8](#_Toc179625992)

[**Supplementary Table S3**. Number individuals with RA enrolled in NEIAA during consecutive 3-month study periods, and the proportion who were escalated to b/tsDMARDs within 12 months of initial rheumatology assessment. 9](#_Toc179625993)

[**Supplementary Table S4**. DAS28 scores at 12 months following initial assessment in individuals with RA who were not escalated to b/tsDMARDs. 10](#_Toc179625994)

[**Supplementary Table S5**. Associations between hospital-level factors and the proportion of individuals with RA within each hospital who were escalated to b/tsDMARDs within 12 months of initial rheumatology assessment. 11](#_Toc179625995)

## **Supplementary Figure S1.** Flowchart of study cohorts.


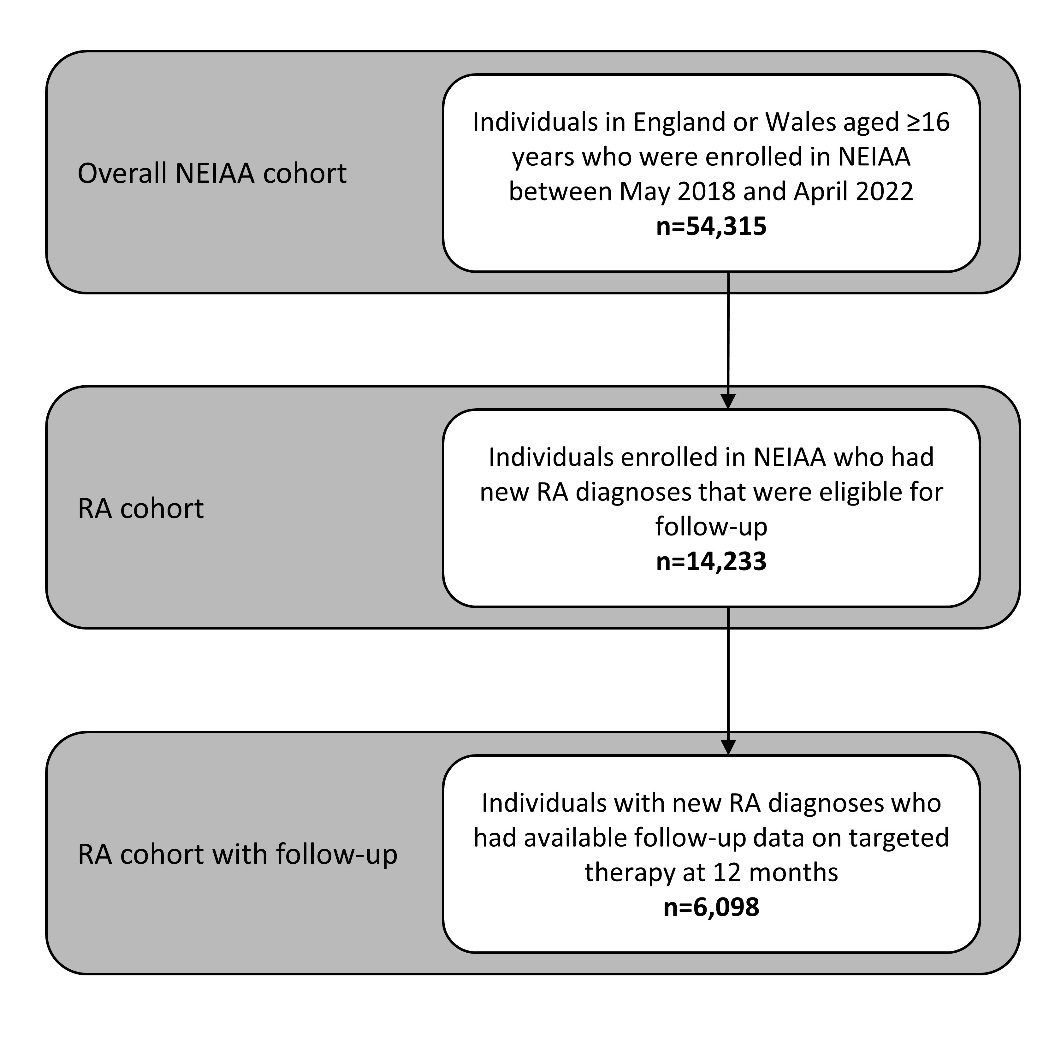


NEIAA: National Early Inflammatory Arthritis Audit. RA: Rheumatoid arthritis.

## **Supplementary Figure S2.** Temporal trends in the proportion of individuals with RA who were commenced on TNF inhibitors vs. other mode-of-action b/tsDMARDs at 12 months after the initial rheumatology assessment.


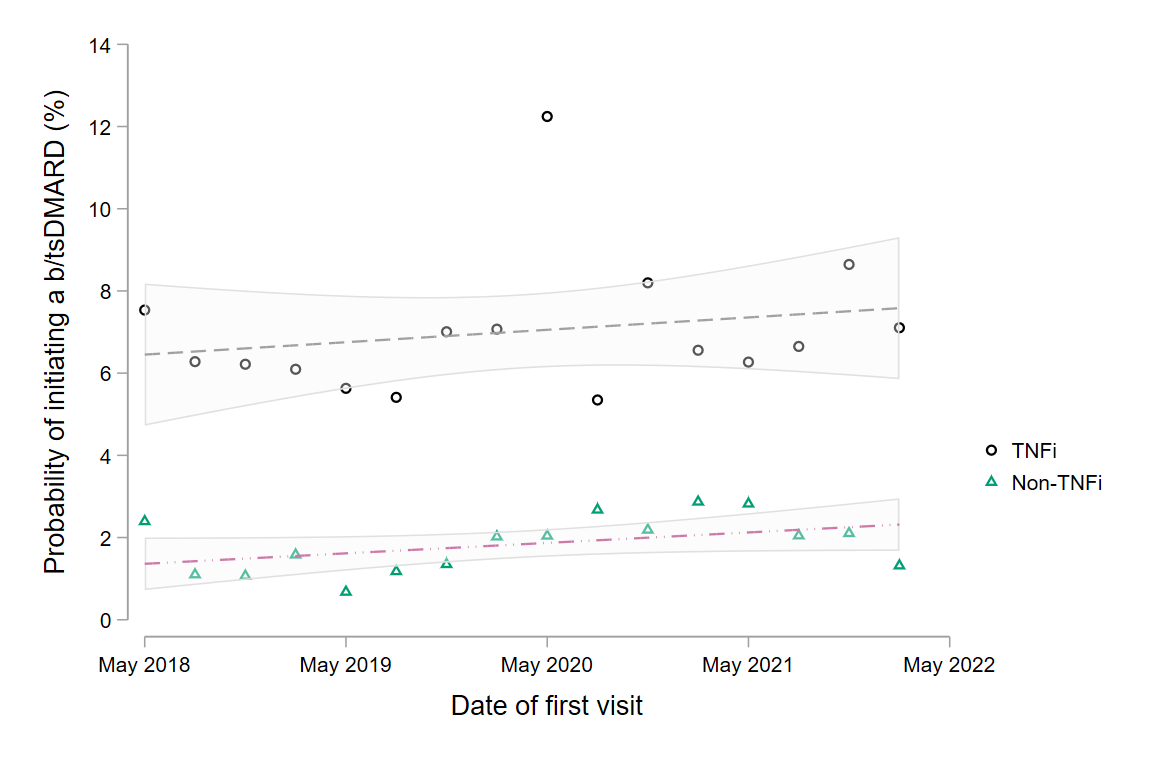


Single time-points represent the mean proportion of individuals escalated to b/tsDMARDs (shown separately for TNF inhibitors [TNFi] and other mode-of-action b/tsDMARDs [non-TNFi]), averaged over 3-monthly time-periods. Linear trend lines are shown with corresponding 95% confidence intervals (shaded areas).

## **Supplementary Figure S3.** Regional variation in the proportion of individuals in NEIAA with new RA diagnoses who were escalated to b/tsDMARDs within 12 months of initial rheumatology assessment.

A


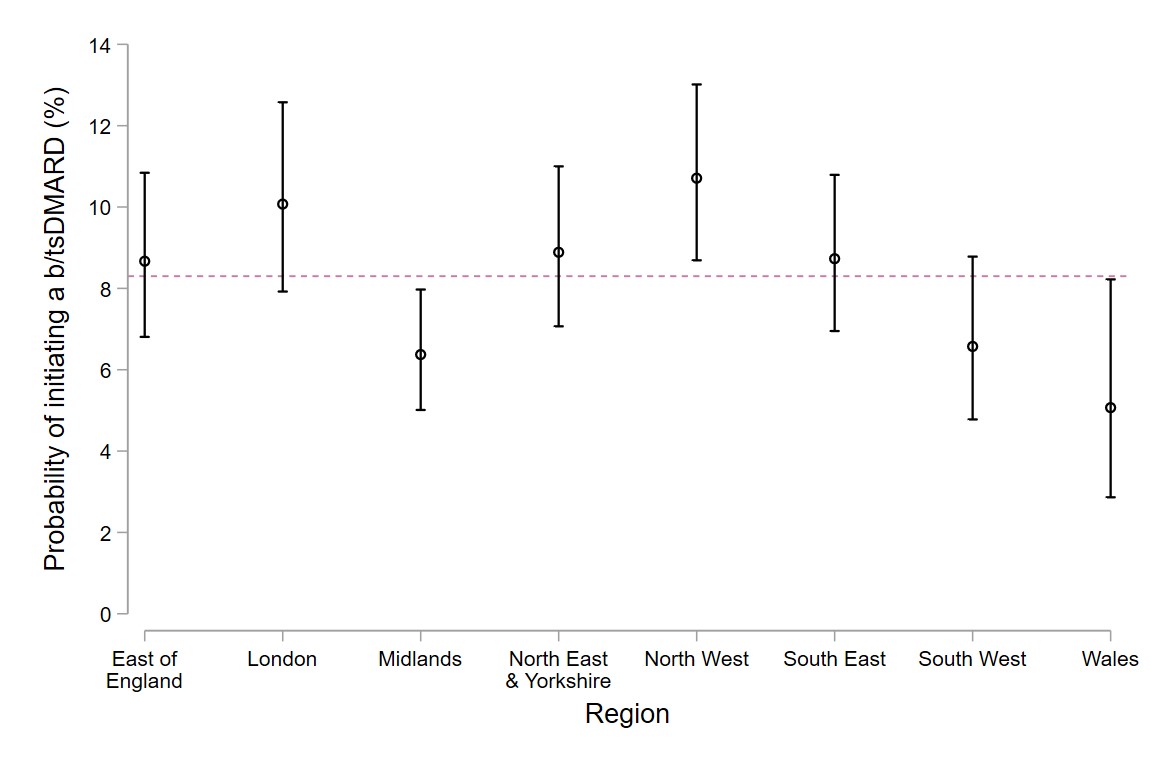

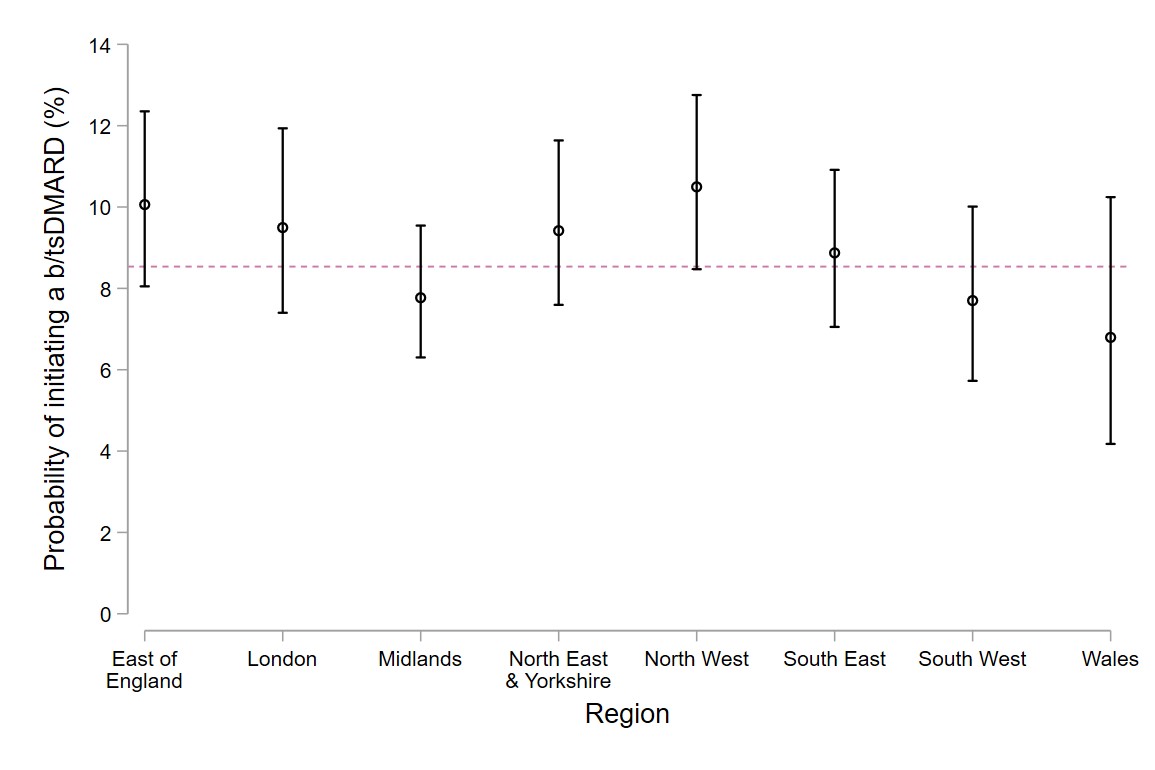


B

Data are shown by region of England and Wales before (*Panel A*) and after case-mix adjustment (*Panel B*), with 95% confidence intervals. Case-mix adjustment was performed using the following variables held at constant levels (specified in brackets) across regions: age (50 years); sex (female); index of multiple deprivation (middle tertile); rheumatic disease comorbidity index (one comorbidity); DAS28 at baseline (5.1). The horizontal dashed line in Panel A represents the observed proportion of individuals who were escalated to b/tsDMARDs, averaged across all regions. In Panel B, the horizontal dashed line represents the expected probability of b/tsDMARD initiation following case-mix adjustment.

## **Supplementary Figure S4.** Temporal and regional variation in the proportion of individuals with RA who were escalated to b/tsDMARDs within 12 months.


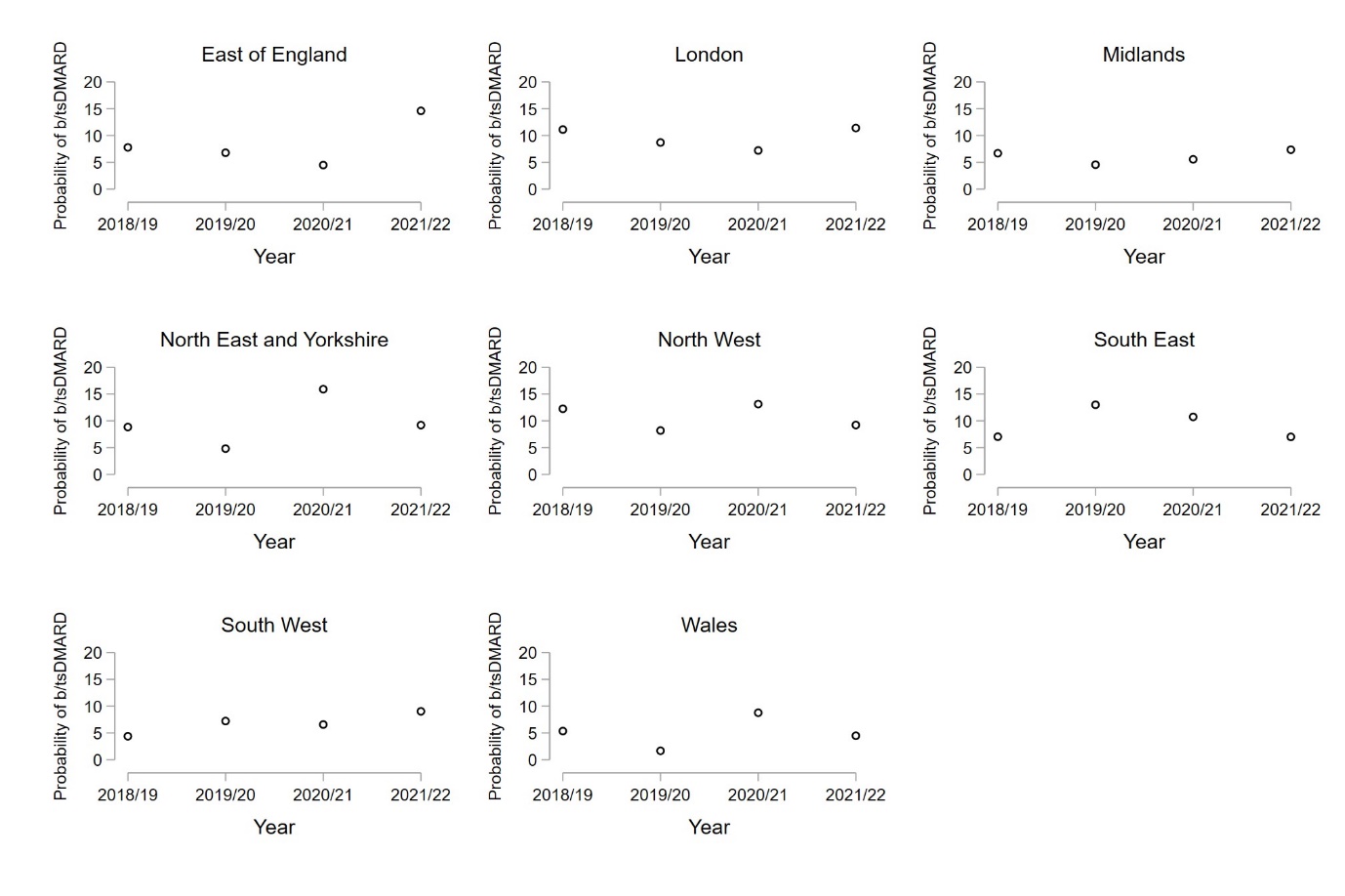


Each figure represents the observed proportion of individuals with new RA diagnoses who initiated b/tsDMARDs within 12 month of initial rheumatology assessment, separated by region and year of initial assessment.

## **Supplementary Figure S5.** Regional variation in the proportion of individuals initiating b/tsDMARDs within 12 months of initial rheumatology assessment who were prescribed TNF inhibitors.

A


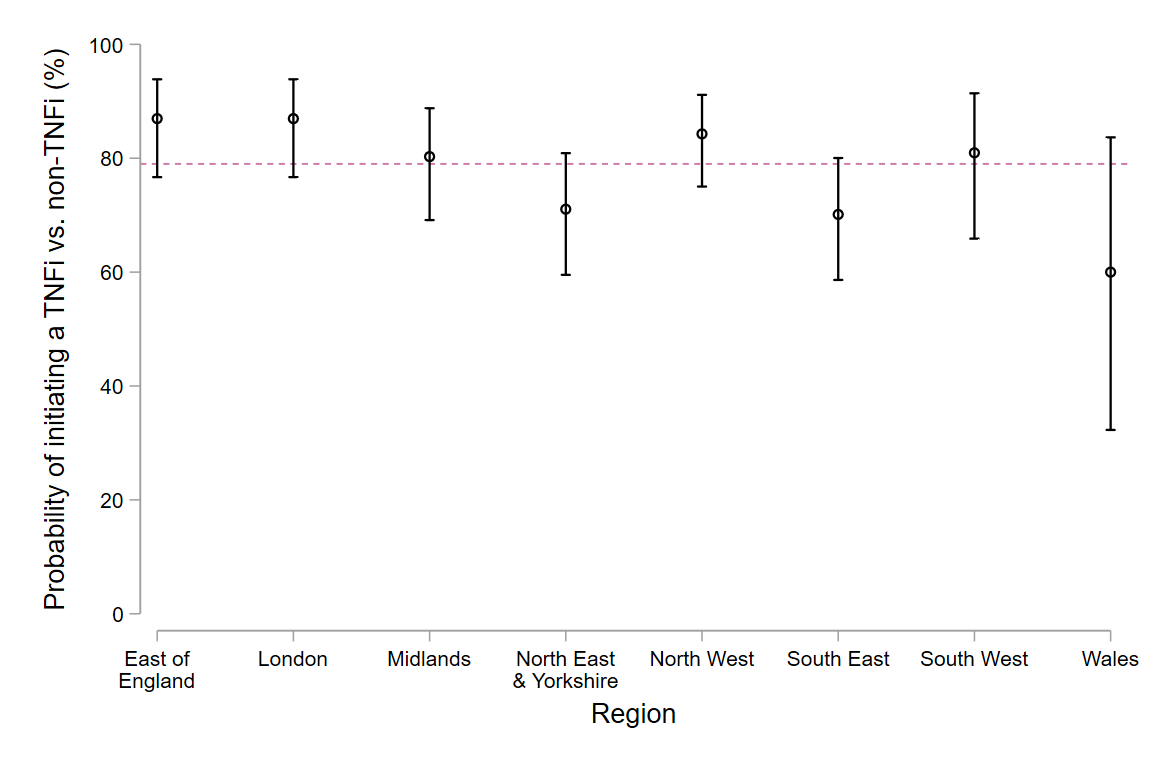

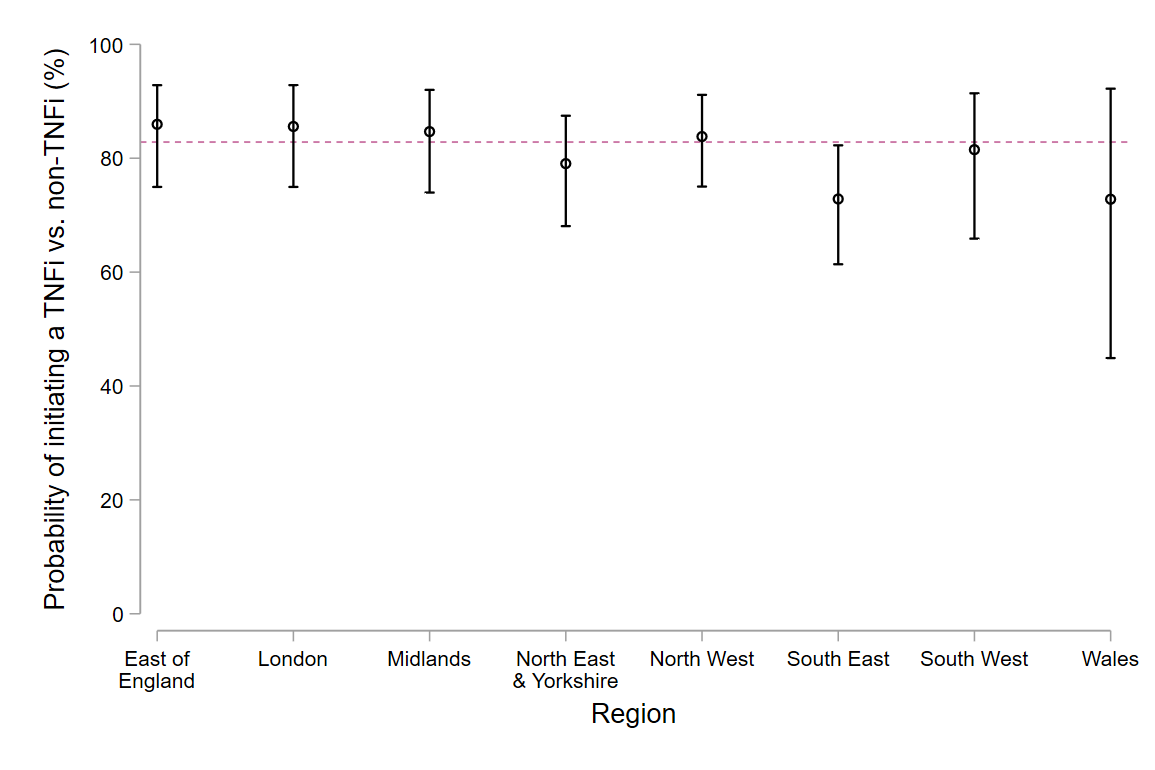


B

The probability of TNF inhibitor use at 12 months for individuals who commenced b/tsDMARDs is shown before (*Panel A*) and after case-mix-adjusted estimates (*Panel B*), with 95% confidence intervals. Case-mix adjustment was performed using the following variables held at constant levels (specified in brackets) across regions: age (50 years); sex (female); index of multiple deprivation (middle tertile); rheumatic disease comorbidity index (one comorbidity); DAS28 at baseline (5.1). The horizontal dashed line in Panel A represents the proportion of individuals who were prescribed TNF inhibitors, averaged across all regions. In Panel B, the horizontal dashed line represents the expected probability of TNF inhibitor use following case-mix adjustment.

## **Supplementary Table S1**. Baseline characteristics of individuals with RA enrolled in NEIAA who had available 12-month follow-up data, compared with individuals with RA without available follow-up data.

|  | **Total** | **12m data available** | **12m data unavailable** |
| --- | --- | --- | --- |
|  | **N=14,233** | **N=6,098** | **N=8,135** |
| Age, years (mean [SD]) | 59.4 (15.5) | 59.2 (14.9) | 59.5 (15.9) |
| Sex |  |  |  |
| Male | 5,194 (36.5%) | 2,186 (35.8%) | 3,008 (37.0%) |
| Female | 9,039 (63.5%) | 3,912 (64.2%) | 5,127 (63.0%) |
| Ethnicity |  |  |  |
| White | 12,117 (85.1%) | 5,215 (85.5%) | 6,902 (84.8%) |
| Black, Asian, Mixed or Other | 1,953 (13.7%) | 832 (13.6%) | 1,121 (13.8%) |
| Not known | 163 (1.1%) | 51 (0.8%) | 112 (1.4%) |
| IMD decile |  |  |  |
| 1-3 (least deprived) | 3,809 (26.8%) | 1,563 (25.6%) | 2,246 (27.6%) |
| 4-7 | 5,565 (39.1%) | 2,457 (40.3%) | 3,108 (38.2%) |
| 8-10 (most deprived) | 4,329 (30.4%) | 1,855 (30.4%) | 2,474 (30.4%) |
| Not known | 530 (3.7%) | 223 (3.7%) | 307 (3.8%) |
| Comorbidity burden (RDCI) |  |  |  |
| None | 7,781 (54.7%) | 3,377 (55.4%) | 4,404 (54.1%) |
| One | 3,084 (21.7%) | 1,331 (21.8%) | 1,753 (21.5%) |
| Two or more | 3,230 (22.7%) | 1,380 (22.6%) | 1,850 (22.7%) |
| Not known | 138 (1.0%) | 10 (0.2%) | 128 (1.6%) |
| Lung disease |  |  |  |
| No | 12,447 (87.5%) | 5,387 (88.3%) | 7,060 (86.8%) |
| Yes | 1,648 (11.6%) | 701 (11.5%) | 947 (11.6%) |
| Not known | 138 (1.0%) | 10 (0.2%) | 128 (1.6%) |
| Cardiovascular disease |  |  |  |
| No | 13,260 (93.2%) | 5,754 (94.4%) | 7,506 (92.3%) |
| Yes | 835 (5.9%) | 334 (5.5%) | 501 (6.2%) |
| Not known | 138 (1.0%) | 10 (0.2%) | 128 (1.6%) |
| Cancer |  |  |  |
| No | 13,513 (94.9%) | 5,846 (95.9%) | 7,667 (94.2%) |
| Yes | 582 (4.1%) | 242 (4.0%) | 340 (4.2%) |
| Not known | 138 (1.0%) | 10 (0.2%) | 128 (1.6%) |
| Depression |  |  |  |
| No | 13,031 (91.6%) | 5,656 (92.8%) | 7,375 (90.7%) |
| Yes | 1,064 (7.5%) | 432 (7.1%) | 632 (7.8%) |
| Not known | 138 (1.0%) | 10 (0.2%) | 128 (1.6%) |
| Smoking status |  |  |  |
| Current smoker | 2,767 (19.4%) | 1,189 (19.5%) | 1,578 (19.4%) |
| Ex-smoker | 4,195 (29.5%) | 1,820 (29.8%) | 2,375 (29.2%) |
| Never smoked | 6,168 (43.3%) | 2,652 (43.5%) | 3,516 (43.2%) |
| Not known | 1,103 (7.7%) | 437 (7.2%) | 666 (8.2%) |
| RhF or CCP positive |  |  |  |
| No | 3,788 (28.4%) | 1,546 (26.4%) | 2,242 (29.9%) |
| Yes | 9,555 (71.6%) | 4,308 (73.6%) | 5,247 (70.1%) |
| Not known | 890 (6.3%) | 244 (4.0%) | 646 (7.9%) |
| DAS28 at baseline (median [IQR]) | 5.0 (4.0,5.9) | 5.1 (4.1,6.0) | 4.9 (3.9,5.9) |
| Not known | 1,008 (7.1%) | 265 (4.3%) | 743 (9.1%) |

IMD: index of multiple deprivation; RDCI: rheumatic disease comorbidity index; RhF: rheumatoid factor; CCP: cyclic citrullinated peptide; DAS28: Disease Activity Score at 28 joints.

## **Supplementary Table S2**. Baseline characteristics of individuals with RA who initiated b/tsDMARDs within 12 months of initial rheumatology assessment, compared with individuals who did not initiate b/tsDMARDs.

|  | **Total** | **b/tsDMARD initiated** | **No b/tsDMARD** |
| --- | --- | --- | --- |
|  | **N=6,098** | **N=508** | **N=5,590** |
| Age, years (mean [SD]) | 59.2 (14.9) | 54.3 (15.7) | 59.6 (14.7) |
| Sex |  |  |  |
| Male | 2,186 (35.8%) | 162 (31.9%) | 2,024 (36.2%) |
| Female | 3,912 (64.2%) | 346 (68.1%) | 3,566 (63.8%) |
| Ethnicity |  |  |  |
| White | 5,215 (86.2%) | 436 (87.0%) | 4,779 (86.2%) |
| Black, Asian, Mixed or Other | 832 (13.8%) | 65 (13.0%) | 767 (13.8%) |
| Not known | 51 | 7 | 44 |
| IMD decile |  |  |  |
| 1-3 (least deprived) | 1,563 (26.6%) | 118 (23.9%) | 1,445 (26.9%) |
| 4-7 | 2,457 (41.8%) | 206 (41.7%) | 2,251 (41.8%) |
| 8-10 (most deprived) | 1,855 (31.6%) | 170 (34.4%) | 1,685 (31.3%) |
| Not known | 223 | 14 | 209 |
| Comorbidity burden (RDCI) |  |  |  |
| None | 3,377 (55.5%) | 298 (58.8%) | 3,079 (55.2%) |
| One | 1,331 (21.9%) | 105 (20.7%) | 1,226 (22.0%) |
| Two or more | 1,380 (22.7%) | 104 (20.5%) | 1,276 (22.9%) |
| Not known | 10 | 1 | 9 |
| Lung disease |  |  |  |
| No | 5,387 (88.5%) | 443 (87.4%) | 4,944 (88.6%) |
| Yes | 701 (11.5%) | 64 (12.6%) | 637 (11.4%) |
| Not known | 10 | 1 | 9 |
| Cardiovascular disease |  |  |  |
| No | 5,754 (94.5%) | 487 (96.1%) | 5,267 (94.4%) |
| Yes | 334 (5.5%) | 20 (3.9%) | 314 (5.6%) |
| Not known | 10 | 1 | 9 |
| Cancer |  |  |  |
| No | 5,846 (96.0%) | 493 (97.2%) | 5,353 (95.9%) |
| Yes | 242 (4.0%) | 14 (2.8%) | 228 (4.1%) |
| Not known | 10 | 1 | 9 |
| Depression |  |  |  |
| No | 5,656 (92.9%) | 460 (90.7%) | 5,196 (93.1%) |
| Yes | 432 (7.1%) | 47 (9.3%) | 385 (6.9%) |
| Not known | 10 | 1 | 9 |
| Smoking status |  |  |  |
| Current smoker | 1,189 (21.0%) | 115 (24.3%) | 1,074 (20.7%) |
| Ex-smoker | 1,820 (32.1%) | 131 (27.6%) | 1,689 (32.6%) |
| Never smoked | 2,652 (46.8%) | 228 (48.1%) | 2,424 (46.7%) |
| Not known | 437 | 34 | 403 |
| RhF or CCP positive |  |  |  |
| No | 1,546 (26.4%) | 91 (18.6%) | 1,455 (27.1%) |
| Yes | 4,308 (73.6%) | 397 (81.4%) | 3,911 (72.9%) |
| Not known | 244 | 20 | 224 |
| DAS28 at baseline (median [IQR]) | 5.1 (4.1,6.0) | 5.7 (4.8,6.5) | 5.0 (4.0,5.9) |
| Not known | 265 | 18 | 247 |

IMD: index of multiple deprivation; RDCI: rheumatic disease comorbidity index; RhF: rheumatoid factor; CCP: cyclic citrullinated peptide; DAS28: Disease Activity Score at 28 joints.

## **Supplementary Table S3**. Number individuals with RA enrolled in NEIAA during consecutive 3-month study periods, and the proportion who were escalated to b/tsDMARDs within 12 months of initial rheumatology assessment.

| **Time period of initial assessment** | **Number of individuals with RA enrolled in NEIAA during time-period** | **Number who initiated b/tsDMARDs within 12 months of assessment** | **Percentage who initiated b/tsDMARDs** |
| --- | --- | --- | --- |
| May 2018 to Jul 2018 | 836 | 82 | 9.8% |
| Aug 2018 to Oct 2018 | 637 | 46 | 7.2% |
| Nov 2018 to Jan 2019 | 563 | 41 | 7.3% |
| Feb 2019 to Apr 2019 | 443 | 33 | 7.4% |
| May 2019 to Jul 2019 | 444 | 28 | 6.3% |
| Aug 2019 to Oct 2019 | 425 | 28 | 6.6% |
| Nov 2019 to Jan 2020 | 371 | 30 | 8.1% |
| Feb 2020 to Apr 2020 | 198 | 18 | 9.1% |
| May 2020 to Jul 2020 | 49 | 7 | 14.3% |
| Aug 2020 to Oct 2020 | 187 | 15 | 8.0% |
| Nov 2020 to Jan 2021 | 183 | 19 | 10.4% |
| Feb 2021 to Apr 2021 | 244 | 22 | 9.0% |
| May 2021 to Jul 2021 | 319 | 28 | 8.8% |
| Aug 2021 to Oct 2021 | 391 | 34 | 8.7% |
| Nov 2021 to Jan 2022 | 428 | 45 | 10.5% |
| Feb 2022 to Apr 2022 | 380 | 32 | 8.4% |
| **Overall** | **6098** | **508** | **8.3%** |
|  |  |  |  |

Data shown are for individuals enrolled in NEIAA with new diagnoses of RA who had follow-up data available on whether they commenced b/tsDMARDs within 12 months of initial rheumatology assessment.

## **Supplementary Table S4**. DAS28 scores at 12 months following initial assessment in individuals with RA who were not escalated to b/tsDMARDs.

| **DAS28**  **at 12 months** | **Full study period**  **2018/2022**  **(n=5,590)** | **Initial assessment**  **2018/2019**  **(n=2,277)** | **Initial assessment**  **2019/2020**  **(n=1,334)** | **Initial assessment**  **2020/2021**  **(n=600)** | **Initial assessment**  **2021/2022**  **(n=1,379)** |
| --- | --- | --- | --- | --- | --- |
| Less than 3.2 | 3,155 (63.4%) | 1,410 (67.0%) | 658 (59.9%) | 331 (61.5%) | 756 (61.3%) |
| 3.2 to 5.1 | 1,391 (28.0%) | 545 (25.9%) | 323 (29.4%) | 151 (28.1%) | 372 (30.2%) |
| Greater than 5.1 | 429 (8.6%) | 149 (7.1%) | 118 (10.7%) | 56 (10.4%) | 106 (8.6%) |
| Not known | 615 | 173 | 235 | 62 | 145 |
|  |  |  |  |  |  |

Data are shown overall and separated by year of initial assessment for individuals enrolled in NEIAA with new diagnoses of RA who were not escalated to b/tsDMARDs within 12 months of initial rheumatology assessment. DAS28: Disease Activity Score at 28 joints.

## **Supplementary Table S5**. Associations between hospital-level factors and the proportion of individuals with RA within each hospital who were escalated to b/tsDMARDs within 12 months of initial rheumatology assessment.

| **Variable** | **β-coefficient** | **95% Confidence Intervals** | **P-value** |
| --- | --- | --- | --- |
| Number of annual rheumatology appointments (per 1,000 appointment increase) | -0.030 | -0.29 to 0.23 | 0.82 |
| Number of rheumatology consultants (per 1 WTE staff member increase) | 0.11 | -1.08 to 1.30 | 0.85 |
| Number of rheumatology nurses (per 1 WTE staff member increase) | -1.14 | -2.41 to 0.13 | 0.078 |
| Ratio of rheumatology consultants to annual rheumatology appointments | 3.84 | -9.66 to 17.33 | 0.57 |
| Ratio of rheumatology nurses to annual rheumatology appointments | 1.64 | -12.45 to 15.73 | 0.82 |
| Ratio of rheumatology consultants and nurses to annual rheumatology appointments | 3.01 | -4.80 to 10.83 | 0.44 |
| Presence of dedicated EIA clinics | -2.90 | -8.92 to 3.12 | 0.34 |
|  |  |  |  |

Estimates are from linear regression models, where the outcome represents the proportion of individuals with RA enrolled in NEIAA within each Hospital Trust who were escalated to b/tsDMARDs within 12 months of initial rheumatology assessment. Predictor variables were the number of annual rheumatology appointments that occurred within each Hospital Trust; the number of whole-time equivalent (WTE) rheumatology consultant staff members within each Hospital Trust; the number of WTE rheumatology nurse specialist staff members within each Hospital Trust; the ratio of WTE rheumatology staff members to the number of annual rheumatology appointments in each Hospital Trust; and whether Hospital Trusts had dedicated early inflammatory arthritis (EIA) clinics.
